# Characterization of a Centrifugal Microfluidic Orthogonal Flow Platform

**DOI:** 10.3390/mi13030487

**Published:** 2022-03-20

**Authors:** Michael Shane Woolf, Leah M. Dignan, Scott M. Karas, Hannah M. Lewis, Kevyn C. Hadley, Aeren Q. Nauman, Marcellene A. Gates-Hollingsworth, David P. AuCoin, Heather R. Green, Geoffrey M. Geise, James P. Landers

**Affiliations:** 1 Department of Chemistry, University of Virginia, Charlottesville, VA 22904, USA; lmd4bt@virginia.edu (L.M.D.); sk3ff@virginia.edu (S.M.K.); hml9wn@virginia.edu (H.M.L.); Kch6pr@virginia.edu (K.C.H.); aqn6cd@virginia.edu (A.Q.N.); jpl5e@virginia.edu (J.P.L.); 2 TeGrex Technologies, Charlottesville, VA 22903, USA;; 3 Department of Chemical Engineering, University of Virginia, Charlottesville, VA 22904, USA; mhollingsworth@med.unr.edu (M.A.G.-H.); daucoin@med.unr.edu (D.P.A.); hrg@nevada.unr.edu (H.R.G.); 4 Department of Chemical Engineering, University of Virginia, Charlottesville, VA 22904, USA; geise@virginia.edu; 5 Department of Mechanical Engineering, University of Virginia, Charlottesville, VA 22904, USA; 6 Department of Pathology, University of Virginia, Charlottesville, VA 22904, USA

**Keywords:** microfluidic, centrifugal, embedded membrane, membrane deswelling, orthogonal flow, exponential decay, decay constant

## Abstract

To bring to bear the power of centrifugal microfluidics on vertical flow immunoassays, control of flow orthogonally through nanoporous membranes is essential. The on-disc approach described here leverages the rapid print-cut-laminate (PCL) disc fabrication and prototyping method to create a permanent seal between disc materials and embedded nanoporous membranes. Rotational forces drive fluid flow, replacing capillary action, and complex pneumatic pumping systems. Adjacent microfluidic features form a flow path that directs fluid orthogonally (vertically) through these embedded membranes during assay execution. This method for membrane incorporation circumvents the need for solvents (e.g., acetone) to create the membrane-disc bond and sidesteps issues related to undesirable bypass flow. In other recently published work, we described an orthogonal flow (OF) platform that exploited embedded membranes for automation of enzyme-linked immunosorbent assays (ELISAs). Here, we more fully characterize flow patterns and cellulosic membrane behavior within the centrifugal orthogonal flow (cOF) format. Specifically, high-speed videography studies demonstrate that sample volume, membrane pore size, and ionic composition of the sample matrix significantly impact membrane behavior, and consequently fluid drainage profiles, especially when cellulosic membranes are used. Finally, prototype discs are used to demonstrate proof-of-principle for sandwich-type antigen capture and immunodetection within the cOF system.

## 1. Introduction

### 1.1. Statement of the Problem

Over the last two decades, centrifugal microfluidic systems have proven useful as robust, analytical platforms capable of performing complex liquid handling, while also integrating sophisticated, multistep analytical processes [1,2,3]. One particular advanced unit operation that has garnered occasional interest in the microfluidic community is embedding porous membranes within centrifugal total analysis systems (μTAS) [4,5] and lab-on-a-CD platforms [6,7,8,9]. This infrequent interest has centered largely on two primary membrane functions—centrifugally assisted lateral flow immunoassays (LFI) [10] and in-line filtration of environmental water samples or whole blood, e.g., sediment and bacteria [11,12,13]. Prior art describes the placement of sample chambers near the center of rotation (CoR), ostensibly to minimize the impact of decaying flow rates by maintaining a longer inlet channel. However, those shifting flow patterns have most often been dismissed or downplayed in previous publications, i.e., none describe or predict a constant flow rate—others predict flow rates changes as high as 9%. Likewise, we recently described a membrane-modulated cOF platform for the centrifugal microfluidic integration of enzyme-linked immunosorbent assays (ELISAs) [14]. In that publication, we alluded to observations that flow rates and membrane behavior changed with sample matrix composition and membrane type. To elaborate, during preliminary studies aimed at the development of on-disc ELISA and sandwich-type immunodetection platforms, it was noted that upstream sample preparation dictated the placement of the sample chamber father from the CoR, and nearer to the embedded detection membrane. For example, higher centrifugal forces are required to facilitate elution of fluid from absorbent materials or to enable on-disc separation of plasma from whole blood, which, in turn, influences placement of the sample chamber relative to the CoR. Here, we more fully characterize how placement of the sample chamber closer to the embedded membrane impacts flow patterns within the centrifugal frame of reference and how cellulosic membrane behavior is impacted by changes in sample matrix composition.

### 1.2. Microfluidic Foundations

Microfluidic platforms offer attractive, unconventional approaches that directly address many of the limitations associated with existing standard sample handling and laboratory-based analytical systems [15]. For example, most conventional filtration systems are tethered to centralized laboratories, requiring large-scale benchtop equipment and precluding deployment to the point of collection. In contrast, most existing LFIs and stacked, non-pressure driven orthogonal flow immunoassay (OFI) systems can be utilized at point-of-care (POC), but these platforms rely entirely upon capillary action to drive fluid flow, which introduces a unique set of limitations [16]. Mathematically, two classical models describe capillary-driven fluid transport through porous matrices, e.g., LFI test strips. First, the Lucas–Washburn equation defines the distance traversed by the fluid front (l) under capillary pressure as

(1) $$l\left(t\right)=\sqrt{\frac{\gamma \;r\;cos\theta }{2\eta }t}$$

where γ is the surface tension at the liquid–air interface, r is the effective pore size (radius) of the porous medium (e.g., membrane), θ is the contact angle between the fluid and said porous medium, η is fluid viscosity, and t is time [17,18,19]. Second, Darcy’s law describes the volumetric capillary flow rate as

(2) $$Q=\frac{\kappa \;WH}{\eta \;L}\Delta P$$

where κ is the permeability, WH is the membrane cross-sectional area, L is the membrane length, and ΔP is the pressure change across the porous membrane material [17,18]. Both models dictate that flow rate and total sample transit time in the capillary flow regime are governed by membrane pore size and fluid viscosity. Thus, to achieve realistic assay run times, capillary-based systems (e.g., conventional LFI systems and non-pressure driven OFI systems) are limited to porous media with larger pore sizes and fluid samples that are non-viscous [16].

Furthermore, assay sample volume is often restricted by LFI test cartridge and absorbent wicking pads that rapidly saturate [20,21]. Constraining sample volume limits assay performance in two ways. First, many biofluids and environmental samples exhibit low target concentrations, necessitating analysis of a larger sample volume (possibly on the order of milliliters) to achieve a positive test response [12,22]. Second, viscous samples (e.g., blood and sputum) may require dilution to prevent membrane clogging/fouling and to facilitate timely movement through a cellulosic matrix. Thus, sample volume constraints limit assay sensitivity, especially for viscous, low titer biofluids, and dilute environmental samples [12,22,23].

**Figure 1:**
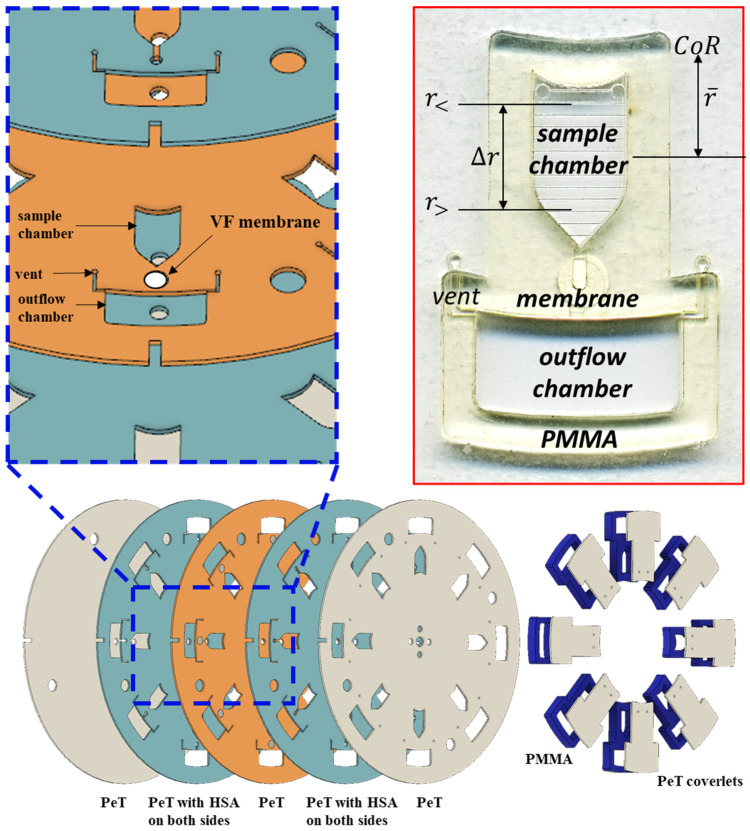
Schematic overview of a the putative cOF disc and a photograph of a single test domain on a fully assembled cOF disc. Diagrammatic exploded view of a six-layer (5 + 1) microfluidic disc design comprised of one polymethyl methacrylate (PMMA) layer and five polyethylene terephthalate (PET) layers. Layers 2 and 4 serve as the primary fluidic layers. Layer 3 functions a flow through or via layer. Circular cutouts (4- or 5-mm Ø) of porous membranes were placed into cutout recesses in layer 3. Upon lamination, HSA coated layers 2 and 4 bond to the membrane, anchoring it in place.

In contrast, the microfluidic platform described here (Figure 1), like the centrifugally driven bioanalytical disc described by Anderson et al. in 1969, Ref. [24], exploits a rotating disc to generate pseudo-gravitational centrifugal forces $\left(f_{\omega }\right)$, defined as

(3) $$f_{\omega }=\rho r\omega^{2}$$

where a liquid body with mass density (ρ), spinning at a frequency (ω), and positioned at a distance (r) from the center of rotation experiences an apparent outward force. In turn, these centrifugal forces generate hydraulic pressure heads ($\Delta p_{\omega }$) and corresponding parabolic flow profiles, with mean fluid velocities ($\bar{\nu }$) given as

(4) $$\Delta p_{\omega }=\rho \bar{r}\Delta r\omega^{2}$$

and

(5) $$\bar{\nu }=\frac{D_{h}^{2}\rho \omega^{2}\bar{r}\Delta r}{32\eta L}$$

where $D_{h}$ is the hydraulic diameter of the channel and $\bar{r}$ and Δr are the average distance of the fluid plug from the CoR and the radial extent of the fluid plug, respectively (Figure 1) [6,7]. Utilization of centrifugal force, as the primary driving force to move fluids within a microfluidic architecture, permits reduction of instrument size and cost by eliminating the need for peripheral hardware, such as pneumatic syringe pumps and the associated tubing networks, while providing precise flow control not possible via passive capillary forces [6,7]. Given the growing body of published literature related to the merits of orthogonal flow immunocapture, Ref. [22], we explored and characterized how embedded membranes might be coupled with on-disc centrifugally driven microfluidic orthogonal flow.

To overcome limitations associated with capillary driven flow, Kainz et al. proposed an on-disc centrifugally assisted LFI system (cLFI) that clearly demonstrated flow regulation through embedded membranes (parallel or lateral flow) via microdevice rotation. The centrifugally assisted LFI system (cLFI) used an architecture where the sample chamber and membrane were placed at radially distant positions from one another [10]. Placing the membrane and sample loading chamber farther apart allowed for a longer inlet channel that, in turn, minimized changes in hydraulic pressure and flow rate (<9%) [10]. This cLFI system, however, was prone to a phenomenon referred to as ‘bypass flow,’ which is undesirable fluid flow over and around the membrane. Accordingly, a mathematical ‘design factor,’ to avoid fluid bypass, was derived. In contrast, Karle et al. and Templeton et al. embedded membranes that were bound to a disc, which reduced or eliminated bypass flow [11,12]. Templeton et al. further demonstrated that insoluble fine particulates and sediments impact flow through embedded membranes, but they did not describe the impact of other solutes in the liquid samples [11].

Here, we report preliminary findings related to our print-cut-laminate (PCL) approach to on-disc orthogonal flow (OF) through embedded, nanoporous membranes (Figure 1) [25]. Collectively, the microfluidic equations described above provided the foundation for these studies and indicated that, within a confined microfluidic frame of reference, and under the influence of a rotational field, centrifugal pumping is impacted by the physical properties of the fluid(s), the radial location of the fluid chambers, channel geometry and orientation, and the rotational frequency of the disc. We empirically characterize and elaborate on some of the more salient talking points underscored in existing publications, including the impact of sample composition on flow, Ref. [11] fluid bypass, Ref. [10] membrane-disc fusion, Refs. [11,12] total time to complete fluid drainage, Ref. [11] and variations in hydraulic pressure head [10].

## 2. Experimental

### 2.1. Fabrication

Each centrifugal OF (cOF) disc was constructed via the print-cut-laminate (PCL) fabrication method [25,26]. Each five-layer disc consisted of alternating sheets of polyethylene terephthalate film (PeT, 101.6 µm thickness, Film Source, Inc., Maryland Heights, MO, USA) and heat sensitive adhesive (HSA, 50.8 µm thickness, EL-7970-39, Adhesives Research, Inc., Glen Rock, PA, USA) [26]. Disc layers, microfluidic architecture, and accessory pieces were designed in AutoCAD^®^ 2019 (Autodesk Inc., San Rafael, CA, USA). 

The microfluidic architecture was ablated into the appropriate layers with a 50 W CO_2_ laser engraving system (VLS3.50, Universal^®^ Laser Systems, Scottsdale, AZ, USA). Layers 2 and 4 served as the primary fluidic layers, layers 1 and 5 acted as capping layers, and the PeT layer 3 functioned as the flow-through or via layer. A disposable biopsy punch was used to obtain circular cutouts (4 or 5 mm Ø) of the porous membranes (Nitrocellulose Membrane, Precut, 0.2 µm pore size, BioRad, Hercules, CA, USA) and polyvinylidene difluoride (Immuno-Blot PVDF, 0.2 µm pore size, BioRad). Membrane cutouts were placed into laser-ablated voids located in layer 3 (4 or 5 mm Ø laser ablated voids spanned the entire thickness of layer 3). To ensure sufficient bonding (sealing) between the membrane and the adjacent disc layers, orthogonal flow ports located in layers 2 and 4 were no larger than 2 mm Ø, i.e., at least 1 mm overlap with the membrane around the circumference. All disc layers were aligned, and heat bonded via a double pass through of an office laminator (~175–190 °C) (UltraLam 250B, Akiles Products, Inc., Mira Loma, CA, USA); disc diameter (Ø) = 70 mm and mean disc thickness = 651 μm. 

Polymethyl methacrylate (PMMA, 1.5 cm, McMaster-Carr, Elmhurst, IL, USA) accessory pieces provided additional chamber depth and volume. For the high-speed video studies, horizontal, linear graduations were rastered into the PeT coverlets of each sample loading chamber (Figure 1). Post-lamination, the PMMA accessory pieces and the concomitant PeT coverlets were bonded to the five-layer disc (Figure 1) with pressure sensitive adhesive (PSA, 55.8 µm thickness, ARcare 7876, Adhesives Research, Inc., Glen Rock, PA, USA). To ensure consistent PSA adhesive bonding, fully assembled cOF discs were pressed under a 10 lb. load for ≥1 h.

### 2.2. Microfluidic Layout and Flow Pattern

Each microfluidic disc consisted of multiple testing domains arranged radially around the disc’s CoR. Each domain was comprised of a sample loading chamber located nearer the CoR, a porous membrane embedded within the middle layer of the disc, and a peripherally located recovery chamber that are all connected by a series of channels (Figure 1). This architecture produced a flow pattern akin to other multilayered fluidic approaches in which a dissolvable film, Ref. [27], laser-valve, Ref. [28], or porous membrane [11,12] is oriented perpendicular to the axis of rotation and embedded between two otherwise open fluidic layers. Disc rotation provided the necessary amount of force to drive fluid radially outward from the sample chamber and through a channel to the orthogonal flow (OF) port. To clarify, the fluidic channel in layer 2 terminates at the OF port, preventing further outward flow, i.e., the only available fluid egress is through the OF port in layer 3 and the embedded membrane. In a rotating frame of reference continuous disc rotation generates sustained hydraulic pressure (Equation (4)). If rotational frequency is sufficiently high, hydraulic pressure within the OF port exceeds the entry pressure of the membrane, forcing fluid to enter and pass through the embedded membrane (transverse or orthogonal flow) [29]. This is akin to fluid flow through standard household plumbing with two 90° turns and an inline filter between; the key differences are scale (micro- vs. macro-) and method of pressure generation. Finally, to flow radially outward from the OF port and into the outlying recovery chamber, the fluid again turned 90°. 

### 2.3. Centrifugally Driven Orthogonal Flow (Proof-of-Principle)

Prior to lamination, 5 mm diameter (Ø) membrane cutouts were inserted into layer 3 of the OF disc. Microfluidic architectures with 1- and 2-mm Ø OF ports were evaluated (Figure 1). To visually characterize flow, aqueous 10 mM erioglaucine dye solution (Sigma-Aldrich, St. Louis, MO, USA) was prepared in artificial blood plasma with bovine serum albumin (0.3341 mM). Artificial blood plasma (ABP, pH = 8.13) was prepared according to Liu et al. and consisted of potassium chloride (0.0131 g KCl), sodium chloride (0.4030 g NaCl), disodium phosphate (0.0086 g Na_2_HPO_4_), sodium bicarbonate (0.0173 g NaHCO_3_), magnesium chloride (0.1545 g MgCl_2_*6H_2_0), and calcium chloride (0.0193 g CaCl_2_) dissolved in 50 mL de-ionized water (diH_2_O) [30]. A 10 µL aliquot of the dye solution was added to each sample loading chamber. The disc was subjected to sequential 30 s spins ranging from 100 to 5500 rpm (500 rpm increments). Images of each disc were captured, as described above. 

### 2.4. Characterization of On-Disc Orthogonal Flow

Fluid samples included assay buffer, artificial urine, and artificial blood plasma. Assay buffer consisted of 0.1 M phosphate buffer (PB) containing 0.1% Triton X-100 and 0.1% bovine serum albumin (BSA, pH = 7.2) (Diagnostics Discovery Laboratory, School of Medicine, University of Nevada, Reno, NV, USA). Artificial urine (AU, pH = 6.77) was prepared according to Liu et al. [31]. The AU solution consisted of urea (1.25 g NH_2_CONH_2_), sodium chloride (0.45 g NaCl), ammonium chloride (0.15 g NH_4_Cl), creatinine (0.1 g C_4_H_7_N_3_O), disodium phosphate (0.125 g Na_2_HPO_4_), monopotassium phosphate (0.125 g KH_2_PO_4_), and sodium sulfite (0.15 g Na_2_SO_3_) dissolved in 50 mL diH_2_O. 

These aqueous simulants were described as the most stable body fluid analogs by Pietryzynska et al. [32]. A 200 µL aliquot of the sample solution was pipetted into a single loading chamber. To visualize the fluid meniscus during this fluid drainage study, the disc was mounted onto a high-speed stroboscopic video system (HSVS) (described below). The disc was then spun at a single rotational frequency (range = 750–2000 rpm in 250 rpm increments) to centrifugally pump the liquid through the OF membrane. Total time to drainage for each 1 mm graduation was recorded. Data exploration, statistical analysis, and visualization were performed in R Studio with the aid of the ggplot2 package (R v.3.5.1 and RStudio v.1.1.456) [33,34,35]. Drainage profiles for six unbacked nitrocellulose membranes were characterized (n = 4 at ea. rotational frequency): BioRad precut nitrocellulose sheets (0.2 µm, BioRad, Hercules, CA, USA), Amersham Protran^®^ and Whatman Protran^®^ BA83/BA85 (0.2 µm and 0.45 µm, GE Healthcare, Little Chalfont, UK), and UniSart^®^ CN140 (0.45 µm, Sartorius, Göttingen, Germany). 

The HSVS was comprised of five major components: a MotionBLITZ EoSens^®^ mini high-speed CMOS recording camera (Mikrotron-GmbH, Unterschleißheim, Germany), MotionBLITZDirector software v.2 1.4.0.1 (Mikrotron-GmbH), a TV ZOOM LENS G6X16 16–100 mm 1:1.9 1” macro (Mikrotron-GmbH), a Nova-Strobe PBL LED portable stroboscope (Monarch Instrument, Amherst, NH, USA), and a custom-built mechatronic spin system that governed disc rotational frequency and strobe rate. The mechatronic spin system consisted of a stepper motor (Sanmotion series, Sanyo denki, Moriguchi, Japan), stepper motor driver (drv8801), photointerrupting optical switch (TT Electronics/Optek Technology, Woking, UK), and an 8-core microcontroller (Propeller P8X32A-M44; Propeller Inc., Rockland, CA, USA).

### 2.5. On-Disc Immunodetection of Ebola Virus-like Particles

The cOF disc was redesigned with a circular, 2 mm port in disc layer 5 (directly below the OF fluidic port). This external port allowed direct, post-lamination access to the exposed cOF membrane, which was spiked with 5 µL of a primary capture antibody solution [10 μg/mL]. After drying (30 min @ 30 °C), each cOF access port was sealed with a 6 mm diameter PeT coverlet—applied using PSA. Positive samples consisted of 40 µL of Ebola virus-like particle antigen [10 µg/mL], 5 µL Au tagged mAb, and 5 µL assay buffer. Negative samples consisted of 5 µL Au tagged mAb and 45 µL assay buffer. Positive and negative samples were added to the sample chamber and allowed to incubate on-disc at room temperature for 15 min prior to flowthrough. The spin protocol used involved 15 s spin cycles at 250 rpm intervals beginning at 1000 and ending at 3000 rpm.

## 3. Results and Discussion

### 3.1. On-Disc Orthogonal Flow

**Figure 2:**
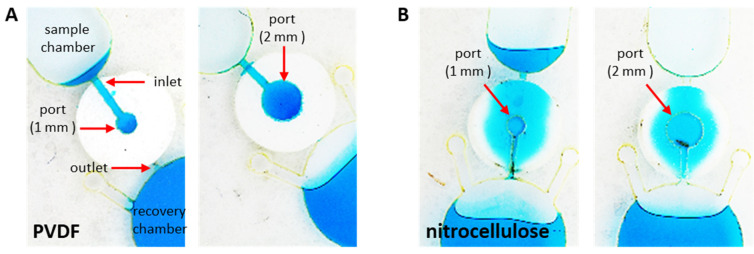
Proof-of-principle dye studies to demonstrate feasibility of disc-based orthogonal flow through porous membranes. Small aliquots (13 µL) of erioglaucine-spiked artificial blood plasma (ABP) were added to each sample chamber to permit visualization of flow pattern through the membrane. Rotational forces were used to pump the fluid aliquots through membranes comprised of (**A**) PVDF and (**B**) nitrocellulose. Fluid intrusion and flow through began at 500 and 4000 rpm, (**A**) and (**B**), respectively. Complete flow through was observed in 30–45 s. This figure features representative AFTER images of the (**A**) PVDF and (**B**) nitrocellulose membranes.

Initial on-disc experiments were conducted to assess the leak-free seal created by the HSA-membrane bond within the microdevice and to demonstrate proof-of-principle continuous, centrifugally driven, orthogonal flow on a PCL fabricated device (Figure 2). Cellulosic materials offered relatively low resistance to flow, i.e., flow was observed when rotational frequency was only slightly above 500 rpm (14× *g* with radial distance from CoR = 5.0 cm). No leaking or bypass flow (seen as dye flow along the perimeter of the membranes) was observed.

As hypothesized, the heat lamination step of the PCL protocol activates HSA that encompasses layers 2 and 4 of the disc, anchoring and sealing the porous membranes into position—coplanar to layer 3. However, this permanent bond between the membrane and adjoining disc layers did not prevent lateral wicking away from the microchannel and OF port (Figure 2B). We hypothesized that radial capillary penetration in the cOF system was inducing fluid movement away from the OF port, and that these regions of lateral flow were secondary to the primary orthogonal flow path defined by the microfluidic architecture. 

In LFI systems, fluid wicking via capillary forces can be understood by considering the Lucas–Washburn equation (Equation (1)) and Darcy’s law (Equation (2)), which describe the extent of radial penetration and volumetric rate of capillary flow, respectively [36,37,38,39,40]. Within an LFI system, as the leading edge of the advancing fluid front crosses the dry region of the porous media, persistent unidirectional capillary flow is sustained via fluid removal by the wicking pad at the distal end of the strip [16,20]. In the context of our disc-based system, it stands to reason that capillary wicking away from the OF port can occur when fluid encounters a dry embedded OF membrane, i.e., liquid is transported away from the primary flow path and into the dry fibrous media via capillary forces. 

Interestingly, the Lucas–Washburn equation presumes relatively dry conditions at the leading edge of the advancing fluid front, and does not consider conditions where the system is partially or fully saturated [36,37,40,41]. We believe that these partially and fully saturated states contribute to LFI flow variability, and significantly limit total allowable LFI sample volume. In contrast, and in the context of the on-disc OF system, we believe that priming (pre-saturating) cellulosic membranes can be employed as a means of limiting radial capillary penetration. 

**Figure 3:**
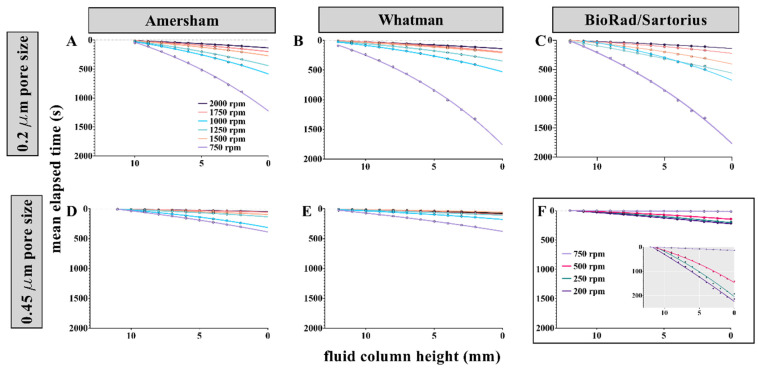
Exponential decay curves depicting the remaining fluid column height in the sample chamber (Δr) as a function of elapsed time. Assay buffer (200 µL) was loaded into each sample chamber (Figure 1 inset). Rotational frequency (rpm) was held constant for each fluid drainage trial (n = 4 for each membrane type-rpm pairing). A high-speed, stroboscopic video system was used to visualize incremental changes in the height of the fluid column over time. Plots (**A**–**F**) depict mean elapsed time (s) vs. the remaining fluid column height in the sample chamber (mm). Colored points represent mean elapsed time for individual trials at each rotational frequency. Solid colored lines represent exponential best fit curves for mean elapsed time values. The top row, plots (**A**–**C**), reflect flow profiles for NC membranes with 0.2 μm pore size. Plots (**D**,**E**) (bottom row) reflect drainage profiles for NC membranes with 0.45 μm pore size. Pore size for the Sartorius Unisart membrane was unknown. Note: Rotational frequencies for Sartorius Unisart membranes (panel (**F**) and (**F**)-inset) were much lower than other membranes (**A**–**E**).

To further assess the disc-membrane seal and to evaluate the extent of radial capillary penetration, the same experiment was repeated using hydrophobic membranes (e.g., PVDF), which showed a dye flow path that was clearly defined by the shape and geometry of the microfluidic architecture (Figure 3A). No net flow was observed at rotational frequencies ≤4000 rpm (894× *g*), suggesting that porous hydrophobic materials offered greater resistance to flow than cellulosic media, thus, requiring higher entry pressures for membrane wetting and initiation of flow [29,42]. No lateral diffusion or capillary penetration away from the primary flow path, leaking along the perimeter of the membranes, or fluid bypass of the membrane were observed [10]. 

These studies showcase several key findings. Porous membranes can be laminated into and sealed within PCL fabricated discs, which creates an effective HSA-membrane bond to prevent fluid bypass and delineate the primary flow path. Orthogonal flow through these embedded porous membranes (hydrophilic and hydrophobic) can be readily controlled via hydraulic pressure generation via disc rotation. Each membrane functions as a pressure sensitive valve, permitting flow only when a sufficient pressure head is achieved [29,42]. Although flow is primarily driven radially outward and dictated by centrifugal forces, when dry cellulosic membranes are used, some radial capillary penetration is to be expected, but this phenomenon may be limited via pre-saturation or priming of the membrane. 

### 3.2. Effect of Membrane Pore Size and Rotational Frequency 

The dye studies discussed above indicate relationships between rotational frequency, membrane type, and fluid entry pressure [29,42]. With this in mind, high-speed video capture was utilized to better understand and characterize the relationships between rotational frequency, total time to drainage, membrane manufacturer, and pore size. Overall, the volumetric flow rate, hereafter referred to as the ‘discharge (*Q*)’, was consistently higher with greater rotational frequencies and with larger membrane pore sizes. The equation for discharge is given as the volume of fluid (V) passing a point in space per unit time (t)

(6) $$Q=\frac{V}{t}=A\frac{\left(distance\right)}{t}=\bar{v}A$$

where Q is proportional to mean fluid velocity ($\bar{v}$) and cross-sectional area of the microfluidic channel (A) [8]. 

Plotting the height of the remaining fluid column (Δr) as a function of elapsed time at a constant rotational frequency revealed a nonlinear decay in the rate of fluid discharge (Figure 3). This nonlinear decay can be understood when considering Equations (4) and (5). When rotational frequency (ω) and fluid mass density (ρ) are held constant, fluid drains from the sample chamber (lower values for in Δr and $\bar{r}$). This decrease in fluid level compels corresponding decreases in the hydraulic pressure ($\Delta p_{\omega }$) applied at the fluid-membrane interface, mean fluid velocity ($\bar{v}$), and fluid discharge (Q) (Equations (4)–(6)). For example, in Figure S1B, if rotational frequency is held constant at 750 rpm (black dots), changes in hydraulic pressure (blue dots) are anticipated as the mean radial extent of the fluid plug in the sample chamber (∆*r*) decreases. These findings are not unlike those observed in classical burette model experiments that were used to empirically determine the value of Euler’s number [43]. That is, the cOF system follows a first order kinetics model in which the rate of fluid drainage is proportional to the amount of fluid remaining in the sample loading chamber; the relationship between total elapsed time and fluid discharge produces continuous curves that take the form of an exponential decay function

(7) $$y=y_{0}e^{-kt}$$

where y is the ending height for the remaining fluid column in the sample chamber, $y_{0}$ is the initial value for the height of the fluid column in the sample chamber, e is Euler’s number, k is the continuous proportionality rate constant, and t is total elapsed time. The magnitude of these k values describe the behavior of the fluid columns undergoing continuous, progressive drainage and is indicative of the rapidity with which that fluid column recedes. Here, the instantaneous rate of change in the height of the fluid column, with respect to time, is proportional to the height of the fluid column itself. 

**Figure 4:**
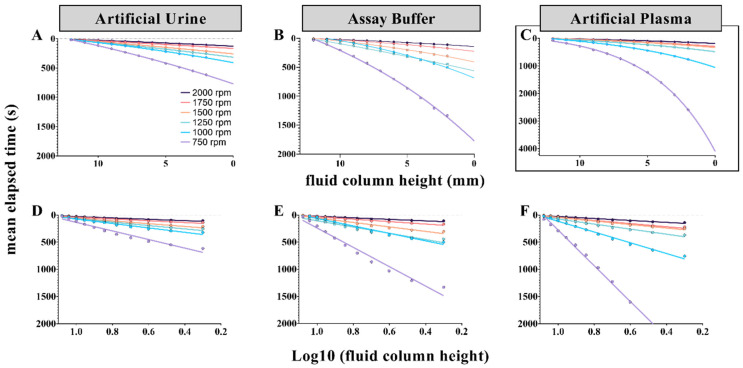
Exponential decay curves depicting the remaining fluid column height in the sample chamber (Δr) as a function of elapsed time. To assess the impact of differing aqueous sample matrices, 4 mm cutouts of BioRAD 0.2 µm pore size membranes were embedded into each cOF disc. Aliquots of each sample solution (200 µL volumes of artificial urine, assay buffer, and artificial blood plasma) were loaded into the sample chambers (Figure 1 inset). Rotational frequency (rpm) was held constant for each fluid drainage trial (n = 4 for each fluid type-rpm pairing). Note: Y-axis for plot C is larger to capture the full extent of the 750 rpm trials. Plots (**A**–**C**) depict mean elapsed time (s) vs. the remaining fluid column height in the sample chamber (mm). Plots (**D**–**F**) demonstrate how those exponential decay curves can be linearized via semilog transformation, i.e., x-axis was transformed to Log10 of the remaining fluid column height.

Semi-log transformation of the *y*-axis linearizes the decay curves (data not shown), revealing the proportionality constant (k) for each relationship. Plots of ln(Δr) as a function of elapsed time are described by the linear equation

(8) $$\ln(\Delta r)=-kt+ln\Delta r_{o}$$

where Δr is the height of the fluid column at time t, $\Delta r_{o}$ is the height of the column at t = 0, and k is the proportionality constant for the draining fluid column (examples of the semi-log plots are featured in Figure 4B,D,F). For all membranes and at all rotational frequencies, a strong, negative correlation between mean elapsed time and ln(Δr) was observed (all $R^{2}$ values exceeded 0.97). Therefore, as the fluid column drains, hydraulic pressure diminishes, and fluid discharge slows. Across all membrane types, larger k values corresponded to higher rotational frequencies and larger pore sizes (Figure 3 and Figure S1), signifying conditions where the fluid column drains more swiftly. Assays requiring continuous flow to measure reaction progress or for assay performance would be adversely impacted by variable fluid drainage rates. We believe that it is possible to remedy issues associated with varying flow speed (drainage rate) by employing a spin protocol that exponentially increases disc rotational frequency throughout the fluid drainage process, thereby maintaining constant hydraulic pressure and flow (Figure S1). 

Some variation in membrane performance was noted between manufacturers and between replicate cuttings from within the same lot of NC membranes (Figure 3), i.e., variation between replicate, raw data points at the same level of fluid drainage (Δr) and at the same rotational frequency, were observed. These variations appeared more pronounced at lower rotational frequencies and as the total elapsed time increased (Figure 3C and Figure 4C). We believe that this variability in the data is best explained by the complex, non-uniform nature of the interconnected three-dimensional (3D) pore networks of the NC membrane cutouts [29,42,44,45]. The size, number, distribution, arrangement, connectedness, and hydrodynamic interaction of pore radii impact hydrodynamic resistance to flow, making some variation in fluidic behavior unavoidable [46]. Simply stated, we do not believe that pore size, as reported by the manufacturer, accurately describes the permeability of these NC membranes, e.g., permeability of the membrane might be different than expected given the reported pore size (Equation (2)) [44,47,48]; It is unlikely that models describing NC membranes as simple bundles of uniformly arranged microtubules adequately characterize membrane complexity or predict fluidic behavior through these cellulosic materials [22,44,49,50].

### 3.3. Effect of Sample Composition 

To assess the potential impact of sample composition on fluid discharge, a second HSVS study was performed. For this study, nitrocellulose (NC) membrane cutouts were taken from a single source (unbacked Whatman BA83 0.2 µm pore size). To reduce the number of contributing variables in this study, we utilized three body fluid ‘simulants’ with viscosities (η) similar to water at room temperature (≅1.0005 cP at 20 °C), namely artificial urine, assay buffer, and artificial blood plasma (Figure 4A–F, respectively). 

Like the HSVS study described in the previous section, plotting the height of the remaining fluid column (Δr) as a function of elapsed time discloses exponential decay curves, which showed that composition of the aqueous system flowing through the porous cOF membranes had considerable impact on fluid discharge. The fluid discharge was greatest when artificial urine was used and least when artificial plasma was used (Figure 4). We hypothesized that these differences were the result of NC fiber swelling; greater fiber swelling would act to restrict flow through the porous network [44]. 

Specific to the fluid drainage profiles presented in Figure 4, the data suggest that the NC fibers may have been most swollen when in contact with the artificial plasma solution and least swollen when in contact with the artificial urine solution. This observed influence of solution composition on fluid drainage rates and its potential connection to NC fiber swelling, can be rationalized by considering the influence of the solutes on the thermodynamic activity of the solvent (water) in the solutions. In general, polymer swelling is restricted as the solute concentration and osmotic pressure of the solution increases, which simultaneously reduces the thermodynamic activity of the solvent [51,52,53]. This phenomenon, often called ‘osmotic de-swelling’, could induce in increased flow through a fibrous network. Stated another way, fiber swelling or de-swelling due to differences in solute concentration alters the microstructure of the porous 3D network and, as described in Equation (5), these changes to membrane permeability (K) influence fluid flow (Q) [10,44]. This description is consistent with the greater fluid discharge observed with artificial urine compared to artificial plasma (Figure 4). Since artificial urine had a greater concentration of solutes than artificial plasma, we would expect less pronounced NC fiber swelling in artificial urine compared to artificial plasma. Moreover, the artificial plasma solution contains sodium bicarbonate—known to promote polymer swelling due to its kosmotropic nature [54,55]. This effect, while perhaps secondary, relative to osmotic de-swelling effects, may also contribute to lessened fluid discharge observed when analyzing artificial plasma. 

**Figure 5:**
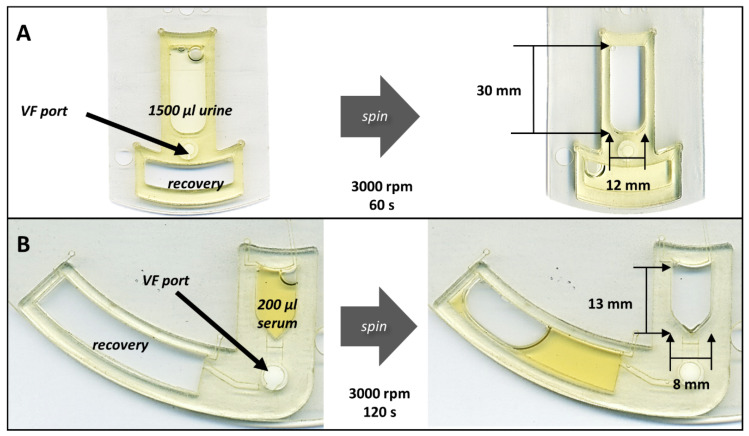
On-disc flowthrough of large volume body fluid samples. (**A**) The cOF disc was redesigned with 4 mm OF port (same radial distance from CoR) and larger chambers, i.e., thicker 1.5 mm PMMA accessory pieces were affixed to the top and bottom of the disc. At 3000 rpm, this cOF design was able to process 1.2 mL of assay buffer (~3 min). Likewise, at 3000 rpm this design processed 1.5 mL of neat urine (<60 s). The bulk fluid was removed from the recovery chamber and two additional 1.5 mL aliquots of urine were passed through the same cOF membrane (total urine volume = 4.5 mL). (**B**) The cOF disc was redesigned with a 4 mm OF port (same radial distance from CoR) and a larger waste chamber. At 3000 rpm, this cOF design processed 200 µL of human serum in ~120 s. Three additional aliquots of serum (200 µL ea) were passed through the same membrane (total serum volume = 800 µL).

The high-speed videography findings presented in Figure 3 and Figure 4 are consistent with the notions that fluid discharge rate is not constant throughout the course of sample processing and that sample composition influences membrane behavior [46,56,57]. While these simulants were not entirely representative of the analogous body fluids, they did provide clear evidence regarding the impact of sample matrix on membrane behavior and flow patterns. Neat (non-artificial) urine and serum may have different viscosities than the analogous artificial simulants. As described in Equation (5), sample viscosity can slow fluid velocity within a microfluidic system. However, when compared to capillary driven systems (Equations (1) and (2)), the impact of sample viscosity on processing time would be greatly diminished in the pressure-driven cOF system. As a good faith demonstration, we have performed additional studies showing that large volumes of neat body fluids can be processed through these embedded membranes (Figure 5). These studies demonstrated that embedded OF membranes can process larger sample volumes, e.g., ≥1.5 mL of urine and ≥800 µL of serum, further experimentation is needed to reduce the footprint of these large volume sample loading chambers, e.g., the sample loading chamber design for the large volume urine handling disc is 12 mm wide and 30 mm long (disc diameter = 70 mm). 

**Figure 6:**
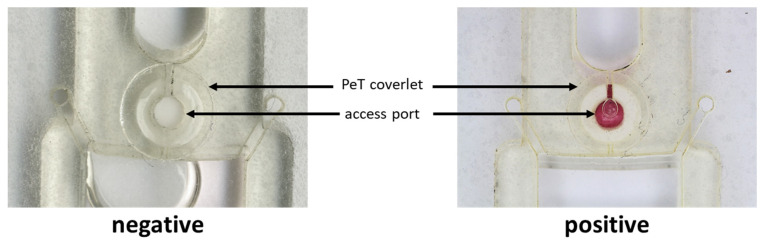
On-disc immunodetection of Ebola virus-like particles (bottom view). The cOF disc was redesigned with circular 2 mm port in disc layer 5 (directly below the OF fluidic port). The exposed cOF membrane was spiked with 5 µL of a primary capture antibody solution [10 ug/mL]. After drying (30 min@30 °C), each cOF access port was sealed with a 6 mm diameter PeT coverlet. Positive samples consisted of 40 µL of Ebola virus-like particle antigen [10 µg/mL], 5 µL Au tagged mAb, and 5 µL assay buffer. Negative samples consisted of 5 µL Au tagged mAb and 45 µL assay buffer. Spin protocol: 15 s spin cycles at 250 rpm intervals beginning at 1000 and ending at 3000 rpm.

Finally, the studies presented thus far in this manuscript provide the necessary insights to exploit embedded membranes for purposes other than simple on-disc filtration, namely an immunoassay [11,12,13]. To achieve this, a cOF disc was fabricated with a small, circular 2 mm port in the bottom PeT layer (5) to allow post-lamination access to the embedded membrane in order to treat each membrane with a primary capture antibody solution. Following drying and curing of the cOF disc and membranes, the access ports can be sealed for subsequent on-disc immunodetection. Figure 6 provides proof-of-principle for this post-lamination approach to membrane functionalization and on-disc immunocapture. Manuscripts detailing the characterization and optimization of the cOF system for pathogen detection (e.g., *Y. pestis* F1 antigen and Ebola virus-like particle) via on-disc sandwich-type immunoassay are imminent; efforts to fully characterize this cOF approach for pathogen detection include blocking and pre-washing (priming) strategies to thwart membrane fouling, preclude loss of biomarker due to non-specific binding, and mitigation of radial capillary penetration. 

## 4. Conclusions

In previous work, we reported a disc-based, membrane-modulated ELISA system [14]. Within that system, nonporous membranes functioned as pressure sensitive, binary flow switches (“on-off”); sample and reaction volumes were small (16–40 μL), allowing incubation, binding, and mixing to occur within the confines of a single chamber without flow through the port. However, some applications and assays will require larger sample volume (e.g., detection of low titer biomarkers or biothreat agents in urine) and some *Sample in*—*Answer out* µTAS devices require on-disc, upstream sample preparation steps (e.g., separation of plasma from whole blood). In those cases, dynamic binding during sample flowthrough is obligatory and it may also be necessary to locate the sample chamber in a more radially outward position, i.e., farther from the CoR and closer to the detection window and porous membrane, which, in turn, increases the potential for significant changes in hydraulic pressure and flow rate. To date, this work represents the first example of empirical characterization of flowthrough within a centrifugal microfluidic system for dynamic binding. More specifically, we have demonstrated the feasibility of on-disc orthogonal flow through unbacked porous membranes, characterized flow decay patterns and membrane behavior within the cOF format, and highlighted the impact of sample composition on transverse flow through the membrane. High-speed videography studies show that, under such conditions, changes in drainage rate due to the diminishing height of the inlet fluid column ($\bar{r}$ and Δr) are quite substantial and take the form of predictable exponential decay curves; in part, these changes are predicted by Equations (4)–(6). To preclude bypass flow (e.g., flow around, not through, the membrane), we describe an embedded membrane system in which the porous media is permanently bonded to the disc via heat lamination. This PCL-based approach to membrane integration creates a leak-free seal between the disc and membrane, directs flow tangentially through the porous media, offers an alternative to other disc-membrane bonding methods, and produces a multilayered flow path akin to those described elsewhere [11,12,27,28]. 

Highspeed videography studies confirmed that sample composition has a remarkable impact on flow through cellulosic membranes. Membrane behavior and flow patterns were significantly influenced by the presence of soluble ionic compounds, changes in solute concentration, osmotic pressure, thermodynamic activity of the solvent (e.g., water), and the kosmotropic effect of bicarbonate [51,52,53,54,55]. We hypothesized that osmotic swelling or de-swelling of the membrane altered the microstructure of porous NC networks, which, in turn, influenced both membrane permeability and influenced fluid discharge rates [10,44]. Although it is unclear how quickly cellulosic membranes swell or deswell, it is possible that longer processing times (longer exposure to the sample matrix) induce more pronounced changes to the porous structure.

Nanoporous cellulosic membranes are standard fare for lateral flow immunoassays and other paper-based microfluidic devices. However, when taken together, the findings reported here suggest that future endeavors to design and optimize any cOF platform should consider membranes that are less sensitive to osmotic swelling. The PCL-based approach described here is robust and capable of integrating other membranes that may be more suitable for other applications; as shown in Figure 2, this cOF platform is amenable to a variety of nanoporous substrates, including polyvinylidene difluoride (PVDF, Bio-RAD), glass microfiber (Whatman GF/a and GF/F), fused silica (Whatman Fusion 5), polyethersulfone (PES), polyacrylonitrile (PAN), and polytetrafluoroethylene (PTFE, Sterlitech). We believe that these proof-of-principle studies highlight critical aspects of on-disc OF prototype development and are the first step toward development of a portable, enclosed, fully integrated system for multiplexed sample processing—including filtration, sample preparation, and immunoassay performance.

## Supplementary Materials

The following supporting information can be downloaded at: https://www.mdpi.com/article/10.3390/mi13030487/s1, Figure S1: Predicted changes in hydraulic pressure head (kPa) as a function of changing fluid column height (Δ*r*).
