# Supplementary material for: Characterization of a Centrifugal Microfluidic Orthogonal Flow Platform
Source: Micromachines (Basel). 2022 Mar 20;13(3):487. doi: 10.3390/mi13030487 (PMC8950265; doi:10.3390/mi13030487)
Supplement: Supplementary file 1 [file micromachines-13-00487-s001.zip › micromachines-1603334-supplementary.pdf]

micromachines

MDPI

Article

Characterization of a Centrifugal Microfluidic Orthogonal Flow Platform

Michael Shane Woolf [1,*], Leah M. Dignan [1], Scott M. Karas [1], Hannah M. Lewis [1], Kevyn C. Hadley [1], Aeren Q. Nauman [1,2], Marcellene A. Gates-Hollingsworth [3], David P. AuCoin [3], Geoffrey M. Geise [4] and James P. Landers [1,5,6]

1 Department of Chemistry, University of Virginia, Charlottesville, VA 22904, USA; lmd4bt@virginia.edu (L.M.D.); sk3ff@virginia.edu (S.M.K.); hml9wn@virginia.edu (H.M.L.); Kch6pr@virginia.edu (K.C.H.); jpl5e@virginia.edu (J.P.L.)
2 TeGrex Technologies, Charlottesville, VA 22903, USA; aqn6cd@virginia.edu (A.Q.N.)
3 Department of Microbiology and Immunology, University of Nevada, Reno, NV 89557, USA; mhollingsworth@med.unr.edu (M.A.G-H.); daucoin@med.unr.edu (D.P.A.)
4 Department of Chemical Engineering, University of Virginia, Charlottesville, VA 22904, USA; geise@virginia.edu
5 Department of Mechanical Engineering, University of Virginia, Charlottesville, VA 22904, USA
6 Department of Pathology, University of Virginia, Charlottesville, VA 22904, USA
* Correspondence: msw2s@virginia.edu

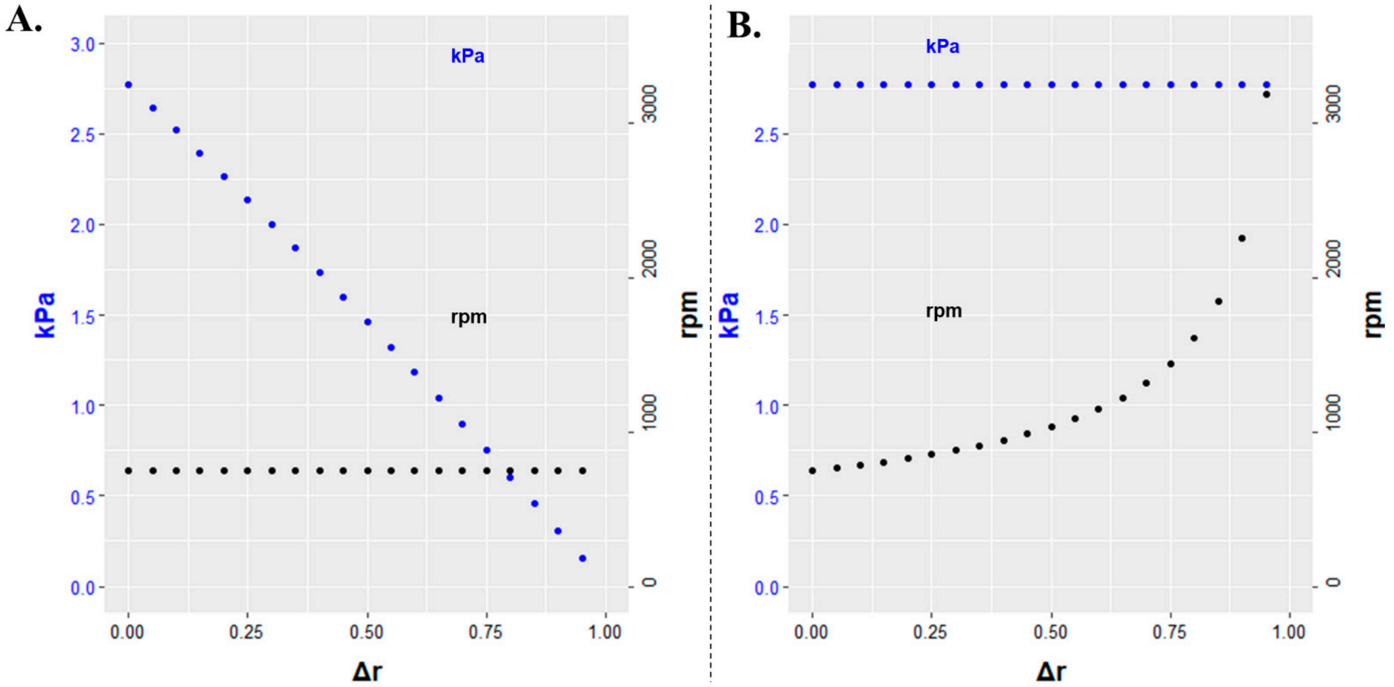

Figure S1. Predicted changes in hydraulic pressure head (kPa) as a function of changing fluid column height (Δr).
